# Structural analysis of full-length SARS-CoV-2 spike protein from an advanced vaccine candidate

**DOI:** 10.1126/science.abe1502

**Published:** 2020-10-20

**Authors:** Sandhya Bangaru, Gabriel Ozorowski, Hannah L. Turner, Aleksandar Antanasijevic, Deli Huang, Xiaoning Wang, Jonathan L. Torres, Jolene K. Diedrich, Jing-Hui Tian, Alyse D. Portnoff, Nita Patel, Michael J. Massare, John R. Yates, David Nemazee, James C. Paulson, Greg Glenn, Gale Smith, Andrew B. Ward

**Affiliations:** 1 Department of Integrative Structural and Computational Biology, The Scripps Research Institute, La Jolla, CA 92037, USA.; 2 Department of Immunology and Microbiology, The Scripps Research Institute, La Jolla, CA 92037, USA.; 3 Department of Molecular Medicine, The Scripps Research Institute, La Jolla, CA 92037, USA.; 4 Novavax, Inc., 21 Firstfield Road, Gaithersburg, MD 20878, USA.

## Abstract

Vaccine efforts to combat the severe acute respiratory syndrome coronavirus 2 (SARS-CoV-2), which is responsible for the current coronavirus disease 2019 (COVID-19) pandemic, are focused on SARS-CoV-2 spike glycoprotein, the primary target for neutralizing antibodies. We performed cryo–election microscopy and site-specific glycan analysis of one of the leading subunit vaccine candidates from Novavax, which is based on a full-length spike protein formulated in polysorbate 80 detergent. Our studies reveal a stable prefusion conformation of the spike immunogen with slight differences in the S1 subunit compared with published spike ectodomain structures. We also observed interactions between the spike trimers, allowing formation of higher-order spike complexes. This study confirms the structural integrity of the full-length spike protein immunogen and provides a basis for interpreting immune responses to this multivalent nanoparticle immunogen.

## Structure of a vaccine candidate

Much effort is being targeted at developing vaccines that will provide protection against severe acute respiratory syndrome coronavirus 2 (SARS-CoV-2). A trimeric spike protein that decorates the virus is a primary target of the host immune system and the focus of vaccine development. Bangaru *et al.* present the structure of a leading vaccine candidate: a full-length spike protein with some modifications aimed at enhancing stability that is formulated in polysorbate 80 detergent. The study confirms that the full-length immunogen is in a stable prefusion conformation and provides a basis for understanding immune responses to the vaccine.

*Science*, this issue p. 1089

## Teaser

Cryo-EM and glycan analysis of a leading SARS-CoV-2 subunit vaccine candidate reveals a stable prefusion conformation of the spike immunogen.

Severe acute respiratory syndrome coronavirus (SARS-CoV) caused a global outbreak from 2002 to 2003 (*1*). Severe acute respiratory syndrome coronavirus 2 (SARS-CoV-2), from the same lineage of the β-CoV genus as SARS-CoV, recently emerged in China and spread rapidly, infecting more than 28 million people worldwide by September 2020 (*2*). Coronavirus disease 2019 (COVID-19), caused by SARS-CoV-2, was declared a pandemic by the World Health Organization (WHO). In response, several SARS-CoV-2 vaccine candidates are being developed and tested at various stages of clinical trials (*3*–*5*). The SARS-CoV-2 spike (S) trimeric glycoprotein is a focus of vaccine development because it is the primary target of host immune defenses (*5*, *6*).

**Fig. 1:**
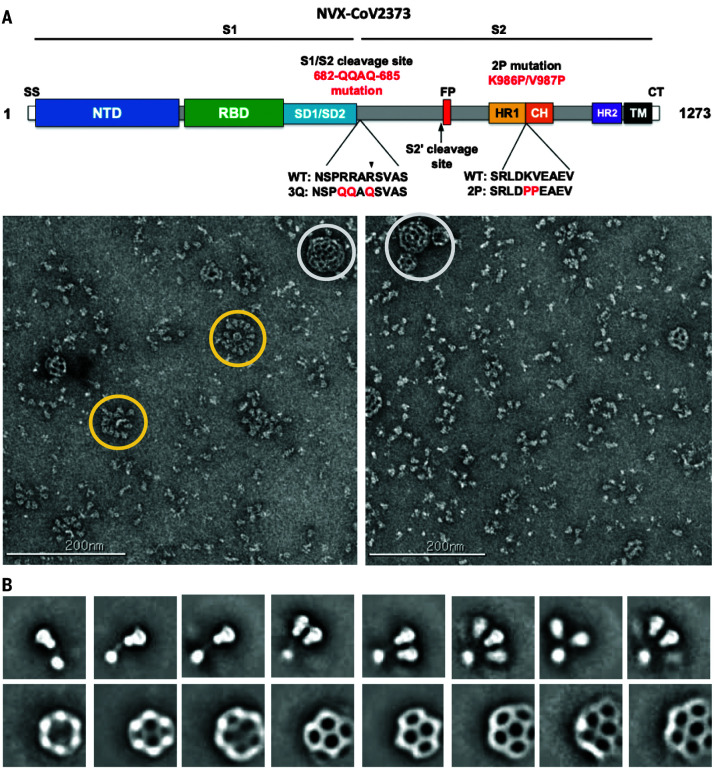
Evaluation of SARS-CoV-2 3Q-2P-FL spike glycoprotein. (**A**) Linear diagram of the sequence and structure elements of the FL SARS-CoV-2 spike protein showing the S1 and S2 ectodomain. Structural elements include a cleavable signal sequence (SS, white), NTD (blue), RBD (green), SD1 and SD2 (light blue), protease cleavage site 2′ (S2′, arrow), fusion peptide (FP, red), heptad repeat 1 (HR1, yellow), central helix (CH, brown), heptad repeat 2 (HR2, purple), TM domain (black), and CT (white). The native furin cleavage site was mutated (RRAR→QQAQ) to be protease resistant and stabilized by introducing two proline (2P) substitutions at positions K986P and V987P to produce SARS-CoV-2 3Q-2P-FL spike. A, Ala; D, Asp; E, Glu; K, Lys; L, Leu; N, Asn; P, Pro; Q, Gln; R, Arg; S, Ser; V, Val. (**B**) Representative negative-stain EM images and 2D classes of SARS-CoV-2 3Q-2P-FL, formulated in PS 80 detergent in the presence of Matrix-M adjuvant. In the raw micrograph, spike rosettes are circled in yellow and Matrix-M adjuvant cages are circled in white. 2D classes showing individual spikes, higher-order spike nanoparticles, and Matrix-M cages of different sizes. Matrix-M does not appear to interact with the spike nanoparticles.

Like other type 1 fusion proteins, the SARS-CoV-2 S prefusion trimer is metastable and undergoes structural rearrangement from a prefusion to a postfusion conformation upon S-protein receptor binding and cleavage (*7*, *8*). The structure of the stabilized SARS-CoV-2 spike ectodomain has been solved in its prefusion conformation and resembles the SARS-CoV spike (*9*–*11*). Here, we describe the structure of a leading SARS-CoV-2 S vaccine candidate (NVAX-CoV2373) based on a full-length (FL) S, residues 1 to 1273, which includes the transmembrane (TM) and the cytoplasmic tail (CT) (Fig. 1A). The final construct, SARS-CoV-2-3Q-2P, was also modified at the S1/S2 polybasic cleavage site from RRAR to QQAQ to render it protease resistant, along with two proline substitutions at residues K986 and V987 in the S2 fusion machinery core for enhanced stability (Fig. 1A). The FL spikes, expressed and purified from insect cells, were formulated in 0.01% (v/v) polysorbate 80 (PS 80) detergent. To characterize the structural integrity of the 3Q-2P-FL immunogen, we performed negative-stain electron microscopy of the FL spike constituted in PS 80 in the presence of Matrix-M adjuvant, recapitulating the vaccine formulation being tested in humans. Imaging revealed trimeric spike proteins present as free trimers or as multitrimer rosettes, containing as many as 14 trimers with their TM domains enclosed in micellar cores of PS 80 detergent (Fig. 1B). Tight clustering of the spikes in the NVAX-CoV2373 nanoparticle formulation may lead to stronger immune responses over soluble trimers alone, similar to other viral glycoprotein immunogens (hemagglutinin and respiratory syncytial virus F) (*12*, *13*).

**Fig. 2:**
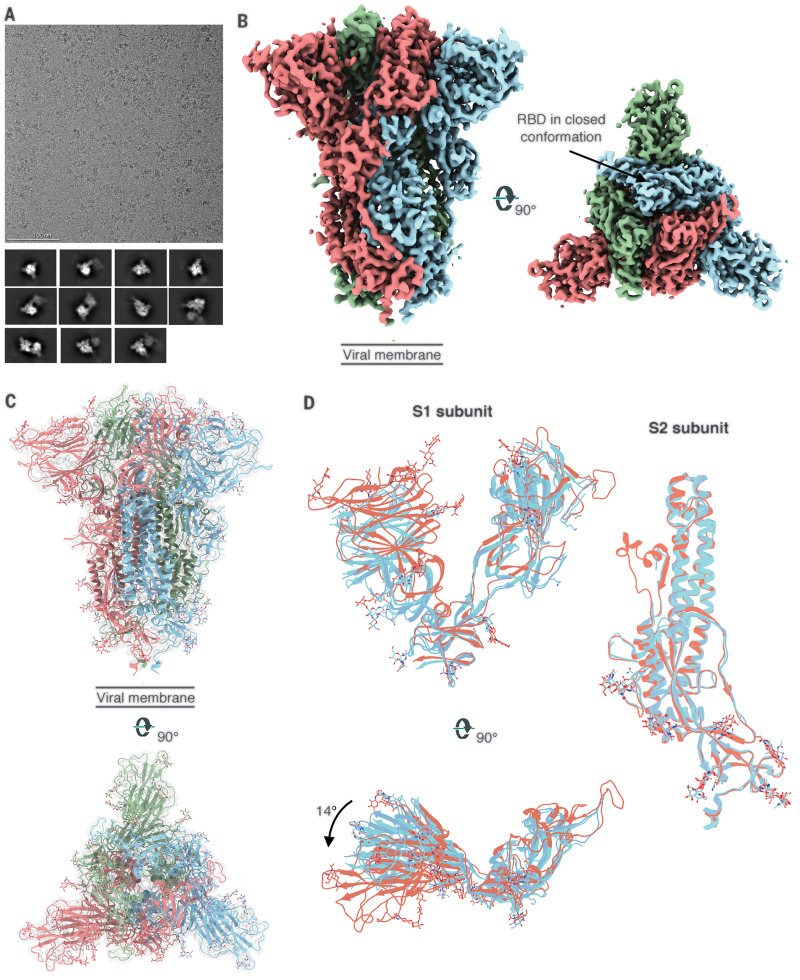
Cryo-EM analysis of SARS-CoV-2 3Q-2P-FL spikes. (**A**) Representative electron micrograph and 2D class averages of 3Q-2P-FL spikes showing free trimers and complexes of trimers. (**B**) Side and top views of the B factor–sharpened cryo-EM map of 3Q-2P-FL free trimers showing the spike in prefusion state, with the RBDs in closed conformation. The protomers are colored in blue, green, and coral for clarity. (**C**) Side and top view of the atomic model of free trimer represented as a ribbon diagram fit into the map density. The protomers are colored in blue, green, and coral, and the map is shown as a transparent gray density. (**D**) Comparison of 3Q-2P-FL spike with published structures (PDB IDs 6VXX and 6VSB) on a subunit level. PDB 6VXX is shown in cyan, PDB 6VSB in blue, and 3Q-2P-FL spike in coral.

We next performed single-particle cryo–electron microscopy (cryo-EM) on the spike formulated in PS 80 detergent (Fig. 2A). Initial two-dimensional (2D) classification revealed the presence of two distinct classes: free spike trimers and dimers of trimers (Fig. 2A). The threefold symmetric (C3) reconstruction of the free spike trimer resulted in a 3.6 Å–resolution map, whereas the asymmetric reconstruction (C1) was refined to 3.8-Å resolution (Fig. 2B and fig. S1, A and B). In previous structures, receptor binding domains (RBDs) exist in either a closed (RBD-down) or an open (RBD-up) conformation that can engage in ACE2 binding (*9*, *10*, *14*). By contrast, we observed that all three RBDs on the 3Q-2P-FL spike trimer were in the closed conformation in our reconstructions (Fig. 2B and fig. S1C). Despite the RBD-down conformation, binding analysis of the 3Q-2P-FL immunogen to ACE2 by both biolayer interferometry and enzyme-linked immunosorbent assay clearly shows binding to ACE2, indicating that the RBD is dynamic and the receptor binding site accessible (*15*). Another study on the prefusion structure of an FL spike protein reported similar findings with RBDs clamped down as a consequence of potential clashes between S2 residues 828 to 853 and subdomain 1 (SD1) when RBD is in open conformation (*16*). Recent reports by Henderson *et al*. have revealed that introducing mutations and removing N-linked glycosylation at certain positions can alter the propensity toward “up” and “down” states of the RBD (*17*, *18*).

**Fig. 3:**
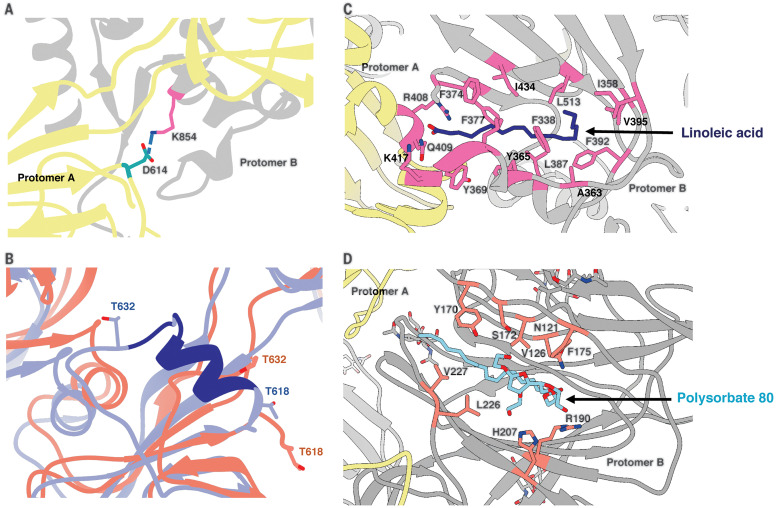
Structural features of the SARS-CoV-2 3Q-2P-FL spike trimer. (**A**) Interprotomeric salt-bridge interaction between D614 and K854 in 3Q-2P-FL spike trimer. (**B**) Comparison of the 615 to 635 loop between 3Q-2P-FL spike shown in coral and PDB 6X6P shown in blue. The residues that were built in 6X6P model but not in our model are shown in dark blue. Threonines at positions 618 and 632 flanking the gap in the 3Q-2P-FL trimer model are shown on both models to highlight their relative positions. T, Thr. (**C**) Linoleic acid (dark blue) binding within a hydrophobic pocket of one RBD where the fatty acid head group reaches out to interact with the closed RBD of the adjacent protomer. The interacting residues are shown in pink. F, Phe; I, Ile; Y, Tyr. (**D**) PS 80 detergent (blue) binding within the NTD with potential hydrogen bonding with R190 and H207. The interacting residues are shown in orange. Adjacent protomers are shown in yellow and gray in (A), (C), and (D). H, His.

Overall, our cryo-EM map was well resolved in both S1 and S2 subunits (fig. S1D), enabling us to model the full S1 N-terminal domain (NTD) and C-terminal domain (CTD) that were less resolved in previous structures (*9*, *10*). Our final atomic model contains residues 14 to 1146 with breaks only in the flexible loop (619 to 631) and the cleavage site (678 to 688) (Fig. 2C). Superimposition of the coordinate models of 3Q-2P-FL spike with published spike structures [Protein Data Bank (PDB) IDs: 6VXX and 6VSB] revealed substantial domain rearrangements in the S1 subunit of 3Q-2P-FL spike (Fig. 2D). The S1 NTD rotated ~14° relative to published models, whereas the CTD and subdomains showed minor local rearrangements (Fig. 2D). Another recent study also observed differences in NTD conformations at lower pH, although our cryo-EM studies were carried out at neutral pH (*19*). In our 3Q-2P-FL structure, we observed a shift in residues flanking the 615 to 635 loop, resulting in a salt bridge between residue D614 on one protomer and K854 on a neighboring protomer (Fig. 3A). This observation is particularly notable given the increased prevalence of D614→G (D614G) mutation in the emerging SARS-CoV-2 strains and its potential role in viral transmission and pathogenesis (*20*). The 615 to 635 loop that is generally disordered in spike trimer structures, including ours, was recently modeled as a helix (PDB ID: 6X6P) (Fig. 3B), although the cryo-EM density (EMD-22078) does not support this assignment (fig. S1E) (*11*).

We observed two additional densities in the S1 subunit that did not correspond to peptide or glycans within the spike (fig. S2A). The first density was buried within a hydrophobic pocket of the CTD (Fig. 3C). We have previously showed palmitoleic acid occupying a similar pocket in the structure of porcine epidemic diarrhea virus (*21*). This density in SARS-CoV-2 S corresponded to linoleic acid, a polyunsaturated fatty acid; the presence of this ligand was confirmed by mass spectrometry of 3Q-2P-FL spike (fig. S2, B and C). The main chain carboxyl group of linoleic acid interacts with the R408 and Q409 residues of the RBD from the adjacent protomer, potentially stabilizing the observed RBD-down state (Fig. 3C) and consistent with a recent report (*22*). The second unassigned density, present in the NTD, was larger and more surface exposed than the first (Fig. 3D and fig. S2D). The aliphatic tail of PS 80 fit well into this hydrophobic pocket, whereas the carbonyl and hydroxyl groups were in proximity to residues R190 and H207 with potential for multiple hydrogen bonds between them (Fig. 3D and fig. S2D). The location of the PS 80 ligand provides a possible explanation for the S1 shift seen in our FL trimer density. PS 80 is specific to the formulation of the Novavax 3Q-2P-FL immunogen, but other ligands may also bind this pocket and provide a potential target for drug design against SARS-CoV-2.

**Fig. 4:**
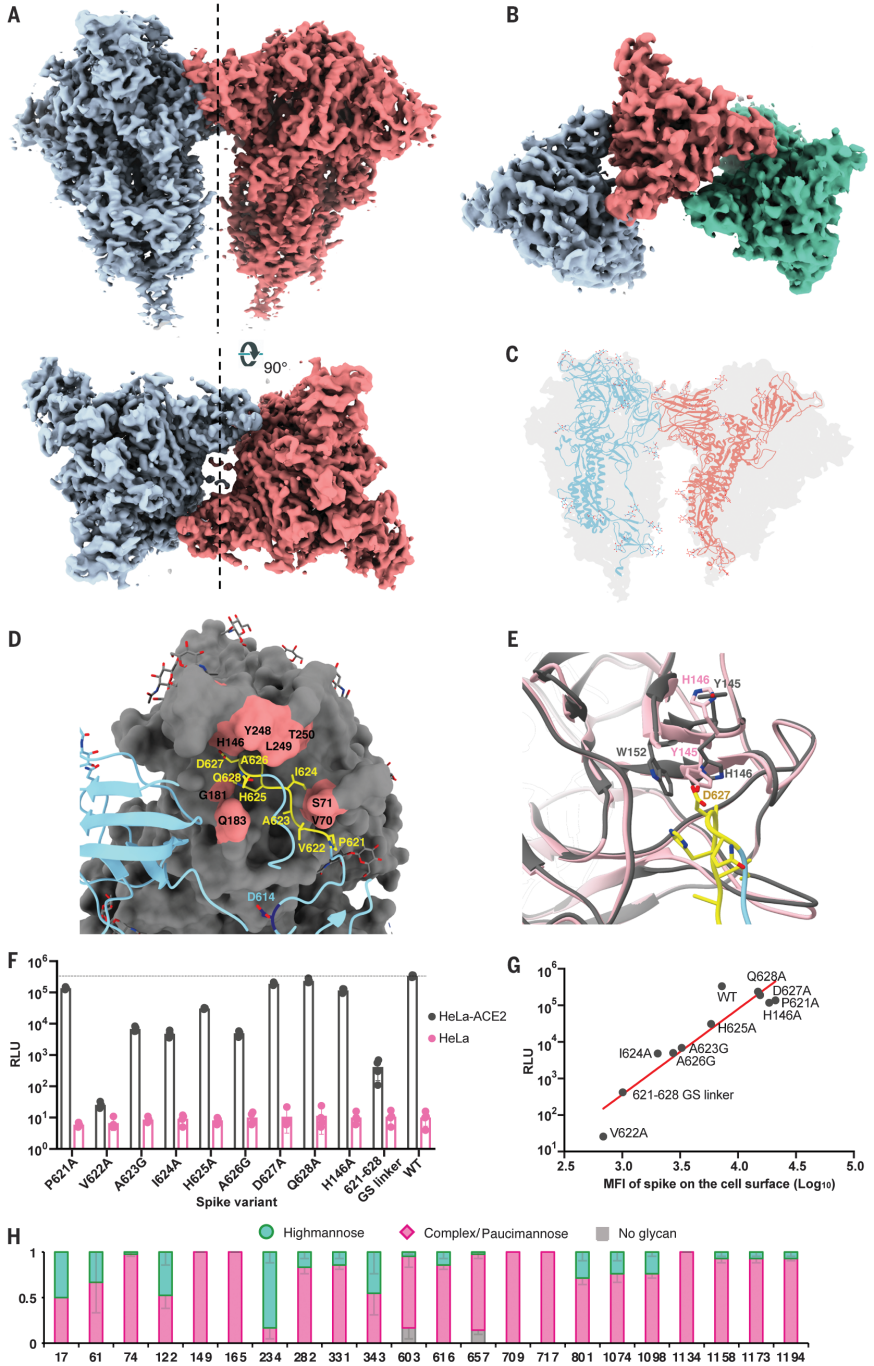
Trimer-trimer interactions and glycan analysis. (**A**) Side and top views of the sharpened cryo-EM map of 3Q-2P-FL dimers of spike trimers. Individual spike trimers are shown in blue and coral along a twofold axis of symmetry (dotted line). (**B**) Top view of the B factor–sharpened cryo-EM map of trimer-of-trimers complex with individual trimers colored in blue, coral, and green. (**C**) Ribbon representation of a protomer from one trimer (blue) interacting with the protomer from the adjacent trimer (coral) docked into the dimers-of-trimers density. (**D**) A close-up view of the interaction between the protomers of adjacent trimers. One protomer is shown as a ribbon diagram in blue, and its binding partner is shown as surface in gray. Residues 621-PVAIHADQ-628 in the loop with potential interactions to the neighboring NTD are colored yellow, and the residues in the NTD binding pocket are highlighted in coral. Residue D614 at the start of the loop is highlighted in dark blue. Glycosylation at residue 616 is not shown for clarity. G, Gly. (**E**) Changes occurring in the binding pocket in the bound state (gray) versus the free trimer (pink). Y145 and H146 switch positions to accommodate the loop better, also resulting in salt-bridge formation between H146 and D627. It also results in stacking between W152 and H146. W, Trp. (**F**) Pseudoviruses expressing SARS-CoV-2 WT or mutant spikes were used to infect HeLa or HeLa-ACE2 cells for 42 to 48 hours. Infection was measured by luciferase intensity RLU (relative light unit) in the lysed cells after infection. (**G**) Correlation between pseudovirus infection (RLU) and surface expression of SARS-CoV-2 spike variants in 293T cells measured by MFI (mean fluorescence intensity). (**H**) Site-specific glycan analysis of 3Q-2P-FL spike protein expressed in Sf9 insect cell line. Proportions shown for no occupancy, oligomannose, and complex or paucimannose potential N-linked glycosylation sites (PNGS) are the average and SEM of 3 to 32 distinctive peptides for each glycosite except for sites 17, 709, and 717, where only a single peptide was observed.

Classification of multimeric spike trimer particles yielded two separate classes: a dimer-of-trimers class that reconstructed to a final resolution of 4.5 Å with twofold symmetry and a trimer-of-trimers class that was resolved to 8.0-Å resolution (Fig. 4, A and B, and fig. S3A). In both reconstructions, the interaction between each pair of trimers involved the SD2 of one protomer from each trimer engaging with the NTD of the adjacent trimer (Fig. 4C), with trimer axes tilted 44.5° relative to each other. The dimer-of-trimer interaction was mainly coordinated by the 615 to 635 loop, which, in contrast to the free-trimer structure, was now fully resolved (Fig. 4D). The loop reaches into and induces subtle changes to a pocket on the adjacent NTD compared with the free-trimer model (Fig. 4D). Residues Y145 and H146 in the binding pocket appear to switch positions in the loop-bound state, resulting in a salt-bridge interaction between H146 and D627 and potential stacking between W152 and H146 (Fig. 4E). We also observed minor displacement of residues 68 to 75 and 248 to 250 surrounding the pocket. In the dimer-of-trimers, we also observed N282 glycans at the dimer interface (fig. S3B). As a control, we also performed cryo-EM studies of the SARS-CoV-2-3Q-FL (without 2P). Notably, the structures of the trimers were identical, and we also observed dimers of trimers (fig. S3, C to E)

Sequence alignment of residues in the 615 to 635 loop and corresponding NTD binding pocket across representative CoV strains belonging to lineage B of betacoronaviruses revealed residues 621-PVAIHADQ-628 are well conserved, but there are notable differences in the binding pocket residues (fig. S4A). Substantial gaps in the interacting NTD loops along with the absence of H146 at the corresponding site on SARS-CoV make it unlikely that SARS-CoV participates in similar intertrimeric interactions. Although the residues in the NTD pocket were almost identical between SARS-CoV-2 and its closely related bat strain Bat-SL-RatG13, we observed some residue differences and one to three amino acid deletions in the loops comprising the NTD binding pocket of representative strains Bat-SL-CoVZC45, BetaCoV/pangolin/Guangdong/1/2019, and BetaCoV/pangolin/Guangxi/P4L/2007 (fig. S4A).

Some human CoVs, including OC43, exclusively use NTD–sialic acid (SA) interactions as their receptor engagement, whereas others such as Middle East respiratory syndrome (MERS) CoV that use the CTD-RBD for primary receptor binding have also been reported to bind SA receptors through their NTD to aid initial attachment to the host cells (*23*–*25*). Structural comparisons of the SARS-CoV-2 NTD dimerization pocket with that of the SA binding site on MERS spike revealed that they did not coincide with each other (PDB ID: 6Q04) (*25*) (fig. S4B). Computational and structural studies have proposed residues on SARS-CoV-2 spike that may be involved in SA binding (*26*, *27*). Structural comparison of this putative glycan binding site to the dimerization site revealed them situated adjacent to one another with residues in loop 70 contributing to both the binding pockets (fig. S4C).

We next performed cell surface expression and pseudovirus replication assays with SARS-CoV-2 wild-type (WT) spike and spikes containing mutations in the 615 to 635 loop and NTD pocket. Each residue in the loop 621-PVAIHADQ-628 and residue H146 in the binding pocket were individually mutated to either alanine or glycine. Additionally, we made a spike construct with all eight residues 621-PVAIHADQ-628 replaced with a glycine-serine (GS) linker to completely abrogate binding. Compared with the WT, the mutants generally exhibited lower levels of infectivity (Fig. 4F). Cell surface expression of these mutants in 293T cells revealed that these mutations also disrupted surface expression of the spike protein, with linear correlation between surface expression and pseudovirus replication (Fig. 4G).

Glycans on viral glycoproteins play a wide role in protein folding, stability, and immune recognition and also in facilitating immune evasion. We therefore conducted site-specific glycosylation analysis of the SARS-CoV-2 prefusion spike protein produced in Sf9 insect cells as previously described (*28*) to assess the extent of glycosylation and the degree of glycan processing from high-mannose or hybrid type to complex type. The analysis detected glycosylation at all 22 N-linked glycan sequons present on SARS-CoV-2 spike (Fig. 4H). Overall, there was high glycan occupancy of >98%, with only two sites (603 and 657) >5% unoccupied. We did not see clear glycan density at either 603 or 657 in the cryo-EM reconstruction of the 3Q-2P-FL spike. Most sites showed extensive glycan processing to complex or paucimannose-type glycans, with only four sites exhibiting ≥40% oligomannose. The glycan analysis also confirmed the presence of glycans at sites 1158, 1173, and 1194 present in the membrane-proximal region of the spike not resolved by cryo-EM. By comparison with site-specific glycan processing of the spike protein produced in mammalian human embryonic kidney (HEK) 293F cells, both mammalian cells and insect cells exhibit extensive processing at most sites. In general, however processing of glycans on the 2019 CoV prefusion spike protein from insect cells was somewhat greater, particularly at sites 709 and 717, which were predominately oligomannose in spike from HEK293 cells but exclusively complex or paucimannose in spike from Sf9 cells (*29*).

Our structural work is consistent with the burgeoning body of spike structures, albeit with notable differences in the rearrangement of S1 domains and formation of intertrimer interactions (*9*, *10*). Both these findings were seen in the FL spike immunogens assembled into compact and dense nanoparticles. Cryo–electron tomographic reconstructions of intact SARS-CoV-2 virions showed a relatively dispersed distribution of spike protein trimers on the viral surface and no evidence of higher-order aggregates (*30*). However, another study showed that the D614G mutation present in close proximity to the dimerization loop results in a several-fold increase of spike numbers on the viral surface, resulting in higher spike protein density and a more infectious virion (*20*). The greater density may be aided by the ability to form such higher-order multimers. Alternatively, the loop that mediates interspike interactions may play a role in viral viability, consistent with our loop mutant data.

Analysis of safety and immunogenicity of the Novavax SARS-CoV-2-3Q-2P-FL immunogen in mice and baboons revealed strong B and T cell responses to the vaccine with no evidence of vaccine-associated enhanced respiratory disease (*15*). Phase 1 and 2 clinical trial results showed that the vaccine induced immune responses exceeding levels seen in COVID-19 patients (*31*). Overall, we found that NVAX-CoV2372 is stable, homogeneous, and locked in the antigenically preferred prefusion conformation. With structural, biophysical, and antigenic characterization now complete, ongoing evaluation in humans will provide the true proof-of-principle for this vaccine concept.

## Supplementary Materials

science.sciencemag.org/content/370/6520/1089/suppl/DC1 Materials and Methods Figs. S1 to S4 Table S1 References (*32*–*52*) MDAR Reproducibility Checklist

## Appendix

View/request a protocol for this paper from *Bio-protocol*.
